# Expanding Community Skin Cancer Services in the UK: Outcomes of Specialist-Led Head and Neck Surgery

**DOI:** 10.7759/cureus.97134

**Published:** 2025-11-18

**Authors:** India Jacklin-Chatha, Ammar Ahsan, Syed Farhan Ahsan, Mohammed Al Abadie

**Affiliations:** 1 Otolaryngology, The Royal Wolverhampton NHS Trust, Wolverhampton, GBR; 2 Dermatology, North Cumbria Integrated NHS Care Foundation, North Cumbria, GBR

**Keywords:** basal cell carcinoma, community dermatology, community skin cancer, head and neck, malignant melanoma, skin cancer, squamous cell carcinoma

## Abstract

Background

Skin cancer incidence is rising in the United Kingdom, increasing pressure on secondary care dermatology and surgical services. Community-based management delivered in local clinic settings outside hospital environments has been proposed to enhance access and efficiency for selected non-melanoma skin cancers. Evidence regarding the safety of community-based surgery for head and neck lesions, which carry a higher functional and cosmetic risk, is limited. This study evaluates outcomes from a specialist-led community head and neck skin cancer surgery service in the West Midlands, UK.

Methods

A single-centre retrospective observational study included all patients undergoing community-based head and neck surgery for suspected skin cancer. Excisions were performed by two head and neck surgeons between January and December 2023. Demographics, lesion histology, anatomical location, margin status, and postoperative outcomes were recorded. Narrow margins were histologically defined as<0.5 cm for all skin cancers. Primary outcomes were completeness of excision, margin involvement, and procedural complications. Postoperative infection was assessed in a randomly selected subset of 30 patients using simple random sampling. Descriptive statistics were used to summarise findings.

Results

A total of 101 patients underwent skin cancer surgery (59.4% male, 40.6% female; median age 75 years, interquartile range 19). Lesions included basal cell carcinoma (BCC) (38.6%, n = 39), squamous cell carcinoma (SCC) (13.9%, n = 14), melanoma in situ (5.0%, n = 5), melanoma (4.0%, n = 4), pre-cancerous lesions including Bowen’s disease and actinic keratoses (5.9%, n = 6), basosquamous carcinoma (2.0%, n = 2), and benign lesions (30.7%, n = 31). Among malignant and pre-cancerous lesions (n = 70), complete excision was achieved in 98.6% (n = 69). One patient with basosquamous carcinoma had an involved margin (1.4%). Ten percent (n = 7) had narrow margins (<0.5 cm). No procedural complications were reported. Among 30 patients randomly selected for postoperative infection assessment, 77% (n = 23) were confirmed negative, and data were unavailable for 23% (n = 7).

Conclusions

Community-based specialist head and neck skin cancer surgery is safe and effective. When performed by appropriately trained professionals under Local Skin Multidisciplinary Team oversight, it achieves high rates of complete excision with minimal complications. Implementing specialist-led community clinics can improve patient access, reduce pressures on secondary care, and provide a scalable model for delivering high-quality skin cancer care closer to patients.

## Introduction

Skin cancer is an increasing public health concern in the United Kingdom. Non-melanoma skin cancers (NMSCs), primarily basal cell carcinoma (BCC) and squamous cell carcinoma (SCC), account for the majority of cases, with approximately 100,000 new diagnoses each year [1]. Although NMSC-related mortality is low, delays in diagnosis and treatment can lead to larger excisions, leading to complex reconstructive procedures and significant functional or cosmetic impairment. Melanoma, while less common, carries higher mortality, with over 13,000 new cases annually and a five-year survival rate of 90% [1].

The rising incidence of skin cancer is placing increasing pressure on healthcare services. In England, the annual costs for skin cancer were estimated at £106-112 million in 2008, with per-case NMSC costs ranging from £889 to £1,226, and projected total expenditures for skin cancer exceeding £180 million by 2020 [2]. In Northern Ireland, total healthcare spending on skin cancer management in 2021-22 was approximately £21 million, primarily driven by melanoma care [3]. According to the July 2025 NHS England Consultant-led Referral to Treatment Waiting Times Data, dermatology services within the NHS remain under significant strain, with only 64% of patients seen within the 18-week target for non-urgent referrals [4], which is compounded by variable referral accuracy in primary care [5]. These challenges underscore the urgent need for strategies that improve timely access while reducing the burden on secondary care.

Community-based management of low-risk NMSCs has been proposed as one such strategy. The British Association of Dermatologists (BAD) provides competency frameworks for community-based practitioners and levels of community skin cancer services. One key group is Model 2 practitioners, which includes general practitioners, nurse specialists, and secondary care specialists, who can manage selected low-risk lesions in the community under the oversight of the Local Skin Multidisciplinary Team (LSMDT). However, complex or high-risk head and neck lesions, particularly those involving critical structures or carrying high cosmetic risk, generally remain outside the independent scope of these practitioners, highlighting the need for specialist-led community services [6]. International experience supports the role of primary care in managing low-risk lesions. In Australia, for example, most skin cancers are managed in primary care, with a large longitudinal study reporting that NMSCs accounted for nearly one-quarter of all skin cancer-related presentations in general practice over 16 years [7].

Evidence regarding community-based excision of head and neck skin cancers remains limited. This study evaluates outcomes from a specialist-led, community-based head and neck skin cancer surgery service to assess clinical safety, completeness of excision, margin status, and postoperative outcomes, and to inform the safe expansion of such services for selected higher-risk lesions.

## Materials and methods

We conducted a single-centre retrospective observational study of patients who underwent community-based head and neck surgery for suspected skin cancer. Excisions were performed by two head and neck surgeons in the West Midlands between January and December 2023. All patient data were anonymised prior to analysis. Patients included were those undergoing surgical excision for suspected skin cancer in the head and neck region during the study period. Patients managed with non-surgical therapies or with recurrent tumours previously treated surgically were excluded. Data collected comprised patient demographics (age and sex), lesion characteristics (histological type and location), margin status, and postoperative outcomes. Narrow margins for all skin cancers were histologically defined as <0.5 cm, representing close but complete excision. This threshold was selected to ensure consistency in reporting across all cases, consistent with thresholds used in recent UK audits of NMSC excision [8]. The LSMDT reviewed all margin-positive and narrow-margin cases for further management planning. Cases requiring wide local excision (WLE), including instances where patients declined WLE, were documented. Anatomical locations were recorded as the central face (cheek, nose, lip, chin, and jawline), upper face (forehead, temple, eyebrow, eyelid, and eye), ear (auricle, periauricular), neck, scalp region (scalp, hairline, and occiput), and the mouth region.

Procedural complications and completeness of excision were recorded for all patients. Postoperative infection was assessed in a randomly selected subset of 30 patients (29.7% of the cohort) using simple random sampling to provide a representative estimate of postoperative infection rates. Primary outcomes were completeness of excision, margin involvement, and postoperative infection. Descriptive statistics were calculated using Microsoft Excel (Version 16.77; Microsoft, Redmond, WA, USA). Age was reported as median with interquartile range (IQR), and categorical variables such as sex, lesion histology, location, and margin status were reported as frequencies and percentages. No formal comparative statistical tests were performed due to the observational nature of the study.

## Results

A total of 101 patients underwent head and neck skin excision surgery during the study period. Of these, 59.4% (n = 60) were male and 40.6% (n = 41) were female. The median age was 75 years (IQR 19; range 24-97). Lesions included BCC (38.6%, n = 39), SCC (13.9%, n = 14), melanoma in situ (5.0%, n = 5), melanoma (4.0%, n = 4), pre-cancerous lesions including Bowen’s disease and actinic keratoses (5.9%, n = 6), basosquamous carcinoma (2.0%, n = 2), and benign lesions (30.7%, n = 31). The central face was the most commonly affected site (33.7%, n = 34), followed by the upper face (22.8%, n = 23), ear (19.8%, n = 20), neck (13.9%, n = 14), scalp and hair-bearing regions (8.9%, n = 9), and the mouth (1.0%, n = 1) (Tables 1, 2). 

**Table 1:** Histological types of head and neck lesions. Data are presented as the number of lesions (N) and the percentage of total lesions (%).

Lesion histology	N	%
Basal cell carcinoma	39	38.6
Squamous cell carcinoma	14	13.9
Pre-cancerous (Bowen’s disease and actinic keratosis)	6	5.9
Melanoma in situ	5	5.0
Melanoma	4	4.0
Basosquamous carcinoma	2	2.0
Benign	31	30.7
Total	101	100.0

**Table 2:** Anatomical distribution of head and neck lesions. Data are presented as the number of lesions (N) and the percentage of total lesions (%).

Anatomical region	Specific sites	N	%
Face – central	Cheek, nose, lip, chin, jawline	34	33.7
Face – upper	Forehead, temple, eyebrow, eyelid, eye	23	22.8
Ear	Auricle, periauricular	20	19.8
Neck	Neck	14	13.9
Scalp and hair-bearing	Scalp, hairline, occiput	9	8.9
Mouth	Mouth	1	1.0
Total		101	100.0

All benign lesions were completely excised. Among malignant and pre-cancerous lesions (n = 70), complete excision was achieved in 98.6% (n = 69). One patient with basosquamous carcinoma had an involved margin (1.4%). Ten percent (n = 7) had narrow margins (<0.5 cm), distributed across BCC (n = 5, 12.8% of BCC cases), melanoma in situ (n = 1, 20% of melanoma in situ cases), and SCC (n = 1, 7.1% of SCC cases) (Tables 3, 4). WLE was performed for all melanomas (n = 4) to achieve oncological clearance, and two additional non-melanoma cases (one SCC, one BCC) required WLE, although one patient declined further surgery. No procedural complications were reported. Among 30 patients randomly selected for postoperative infection assessment, 77% (n = 23) were confirmed negative, and data were unavailable for 23% (n = 7).

**Table 3:** Surgical margin status of malignant and pre-cancerous lesions. Data are presented as the number of malignant or pre-cancerous lesions (N) and the percentage of total malignant/pre-cancerous lesions (%). Narrow margins were defined as <0.5 cm. Fully excised lesions include those with narrow margins.

Margin status	N	%
Fully excised	69	98.6
Involved margin	1	1.4
Narrow margin (<0.5 cm)	7	10.0
Total	70	100.0

**Table 4:** Narrow margins by histology. Data are presented as the number of malignant or pre-cancerous lesions with narrow margins (N) and the percentage of total lesions of that histology (%). Narrow margins were defined as <0.5 cm. BCC, basal cell carcinoma; SCC, squamous cell carcinoma.

Histology	Narrow margins (N)	Total cases (N)	% of Histology cases
BCC	5	39	12.8
Melanoma in situ	1	5	20.0
SCC	1	14	7.1

## Discussion

This study demonstrates that specialist-led community-based head and neck skin cancer surgery is safe and effective, achieving complete excision in 98.6% of cases. Narrow margins were observed in 10% of malignant and pre-cancerous lesions, and no procedural complications or postoperative infections were identified. In the United Kingdom, most NMSCs are initially identified in primary care but traditionally managed in secondary care. Rising incidence, combined with ongoing pressures on NHS dermatology services, contributes to treatment delays and strains hospital resources [4]. Appropriately selected cases managed in community settings may reduce secondary-care burden, freeing resources for more complex cases and potentially improving service efficiency, waiting times, costs, and patient satisfaction. Previous work from our team highlighted that community-based skin cancer surgery can be implemented safely for NMSCs, alleviating pressure on secondary care [9]. This study builds on this by focusing specifically on specialist-led community surgery for head and neck lesions, which present higher functional and cosmetic complexity.

International evidence supports a dual-model approach to skin cancer surgery. In Australia, where the incidence of skin cancer is among the highest globally, primary care plays a central role in management, and the majority of NMSCs are treated within general practice [7,10]. However, evidence from other countries indicates that outcomes vary markedly by practitioner type. A cross-sectional study from the Netherlands of 2,986 BCCs reported complete excision rates of 70% for general practitioners (GPs) compared with 93% for dermatologists and 83% for plastic surgeons, with particularly poor results for head and neck lesions [11]. A 2008 Scottish retrospective analysis showed that GPs, even those with a special interest in skin surgery, achieved inferior outcomes, especially for head and neck lesions [12]. Similarly, a further 2014 Scottish retrospective analysis found that GPs typically excised smaller, low-risk lesions located outside the head and neck and had significantly lower complete excision rates for BCCs than secondary-care specialists [13]. Collectively, these findings suggest that higher-risk or anatomically complex cases, particularly in the head and neck, benefit from specialist oversight.

Further supporting the importance of specialist-led care, a 2004 retrospective observational study of 1,536 patients found that dermatologists achieved superior surgical and survival outcomes for melanoma. When compared with general or plastic surgeons, dermatologists achieved significantly better overall, disease-free, and recurrence-free survival [14]. Such evidence underpins the governance model advocated by the BAD, which emphasises risk stratification and appropriate practitioner expertise for community-based services.

Incomplete excision remains a key surgical quality indicator in NMSC management. The 2020 BAD National Audit on NMSC excision reported a 97% complete excision rate across 3,290 excisions in UK secondary care, with 56.7% of lesions located in the head and neck and a complication rate of <1% [8]. Supporting these findings, a 2016 single-unit study of 2,586 BCCs reported an incomplete excision rate of approximately 7%, with residual tumour present in over half of re-excised specimens [15]. Complementing this, a 2022 national audit of 2,607 NMSC excisions performed by UK plastic surgeons reported incomplete excision rates of 5.3% for BCCs and 7.7% for SCCs, with a higher risk associated with head and neck lesions and recurrent tumours [16]. In our series, the 98.6% complete excision rate compares favourably with these published benchmarks, indicating that specialist-led community services can achieve surgical outcomes comparable to hospital standards. These findings are particularly notable given the higher surgical complexity of head and neck lesions due to anatomical constraints, risk of perineural invasion, and likelihood of recurrence, highlighting the importance of specialist oversight even in community-based services [17].

According to BAD guidance, Model 2 practitioners can manage low-risk lesions, while higher-risk cases, especially those involving the head and neck or cosmetically sensitive sites, require oversight by an LSMDT. Our findings demonstrate that when led by specialists, community-based clinics can safely manage these higher-risk lesions, achieving high complete-excision rates with minimal complications. This supports a dual-model framework in which low-risk lesions are treated by appropriately trained Model 2 practitioners, and higher-risk cases are handled by specialists under structured governance. Such an approach balances safety, efficiency, and accessibility, aligning with national policy to expand community-based skin cancer services without compromising quality.

This study demonstrates that specialist-led community management of head and neck skin cancers can achieve outcomes comparable to hospital standards. While prior UK research has focused on general practitioners, evidence on specialist-led community surgery has been limited. Our findings provide real-world support for the safety, efficacy, and feasibility of expanding specialist-led services within the BAD’s dual-model framework. However, these findings should be interpreted in the context of the single-centre, retrospective design.

Finally, the limitations of this study should be acknowledged. The retrospective design and the single-centre setting involving two surgeons may reduce generalisability to other services. The sample size was modest, and postoperative infection data were available only for a small randomly selected subset. Follow-up was limited, precluding assessment of long-term recurrence and patient-reported outcomes. In addition, the absence of comparative analysis with hospital-based cases restricts broader evaluation of relative effectiveness. Despite these constraints, the study provides valuable real-world evidence supporting the safety and feasibility of specialist-led community-based head and neck skin cancer surgery, offering a foundation for larger, multi-centre prospective studies incorporating formal cost-effectiveness analyses and patient-reported outcome measures to validate and extend these findings.

## Conclusions

Community-based specialist head and neck skin cancer surgery is a safe and effective approach. When performed by appropriately trained professionals under LSMDT oversight, it achieves high rates of complete excision, low complication rates, and excellent outcomes. Implementing specialist-led community clinics can improve patient access and reduce pressures on secondary care, offering a scalable model for delivering high-quality skin cancer care closer to patients.

## References

[1] (2025). NICE Clinical Knowledge Summaries: Skin Cancers - Recognition and Referral. https://cks.nice.org.uk/topics/skin-cancers-recognition-referral/.

[2] Vallejo-Torres L, Morris S, Kinge JM, Poirier V, Verne J (2014). Measuring current and future cost of skin cancer in England. J Public Health (Oxf).

[3] McFerran E, Donaldson S, Dolan O, Lawler M (2024). Skin in the game: The cost consequences of skin cancer diagnosis, treatment and care in Northern Ireland. J Cancer Policy.

[4] (2025). NHS England: Referral to Treatment Waiting Times Data 2025-26. https://www.england.nhs.uk/statistics/wp-content/uploads/sites/2/2025/10/Download-Waiting-Times-by-Hospital-Trust-Aug25-XLS-10M-71939.xls.

[5] Shivakumar H, Chanda UL, Onwuchekwa O (2025). Evaluating the diagnostic accuracy and challenges of the two-week wait referral pathway for skin cancers in primary care. Cureus.

[6] Nasr I, McGrath EJ, Harwood CA (2021). British Association of Dermatologists guidelines for the management of adults with basal cell carcinoma 2021. Br J Dermatol.

[7] Reyes-Marcelino G, McLoughlin K, Harrison C (2023). Skin cancer-related conditions managed in general practice in Australia, 2000-2016: A nationally representative, cross-sectional survey. BMJ Open.

[8] Keith DJ, Bray AP, Brain A (2020). British Association of Dermatologists (BAD) National Audit on Non-Melanoma Skin Cancer Excision 2016 in collaboration with the Royal College of Pathologists. Clin Exp Dermatol.

[9] Ahsan SF, Al Abadie S, Ahsan A, Al Abadie M (2024). Skin cancer surgery in the community: Alleviating the burden on secondary care. Int J Clin Expl Dermatol.

[10] Thompson BS, Pandeya N, Olsen CM, Dusingize JC, Green AC, Neale RE, Whiteman DC (2019). Keratinocyte cancer excisions in Australia: Who performs them and associated costs. Australas J Dermatol.

[11] Ramdas K, van Lee C, Beck S (2018). Differences in rate of complete excision of basal cell carcinoma by dermatologists, plastic surgeons and general practitioners: A large cross-sectional study. Dermatology.

[12] Murchie P, Delaney EK, Thompson WD, Lee AJ (2008). Excising basal cell carcinomas: Comparing the performance of general practitioners, hospital skin specialists and other hospital specialists. Clin Exp Dermatol.

[13] Haw WY, Rakvit P, Fraser SJ, Affleck AG, Holme SA (2014). Skin cancer excision performance in Scottish primary and secondary care: A retrospective analysis. Br J Gen Pract.

[14] McKenna DB, Marioni JC, Lee RJ, Prescott RJ, Doherty VR (2004). A comparison of dermatologists', surgeons' and general practitioners' surgical management of cutaneous melanoma. Br J Dermatol.

[15] Masud D, Moustaki M, Staruch R, Dheansa B (2016). Basal cell carcinomata: Risk factors for incomplete excision and results of re-excision. J Plast Reconstr Aesthet Surg.

[16] Nolan GS, Dunne JA, Lee AE (2022). National audit of non-melanoma skin cancer excisions performed by plastic surgery in the UK. Br J Surg.

[17] Rampinelli V, Pinacoli A, Piazza C (2024). Head and neck nonmelanoma skin cancers: Surgical management and debated issues. Curr Opin Otolaryngol Head Neck Surg.

